# Responding to infectious diseases in Burma and her border regions

**DOI:** 10.1186/1752-1505-2-2

**Published:** 2008-03-14

**Authors:** Chris Beyrer, Thomas J Lee

**Affiliations:** 1 Center for Public Health and Human Rights, Department of Epidemiology, Johns Hopkins Bloomberg School of Public Health, 615 N. Wolfe St., E 7152, Baltimore, MD, 21205, USA; 2 School of Medicine, University of California at Los Angeles, 924 Westwood Blvd, Suite 300, Los Angeles, CA, 90024, USA

## Introduction

In January of 2007 an international scientific conference "Responding to Infectious Diseases in the Border Regions of South and Southeast Asia" was conducted by our collaborative group, and hosted by the Faculty of Tropical Medicine of Mahidol University in Bangkok, Thailand. The conference was something of a landmark, in that it attempted to bring together groups and individuals working on infectious diseases in Burma/Myanmar proper, those working on her border regions, and concerned representatives and scientists from the Burma neighbor states of Thailand, China, India and Bangladesh. Some 190 representatives from 9 countries attended, with representatives from Government, Academia, NGOs, relief groups including MSF France and MSF Switzerland, WHO SEARO Office and representative from WHO and UNAIDS in Burma/Myanmar, the U.S. CDC and USAID, and European donors including DFID. The diseases of concern included HIV/AIDS, TB, malaria, neglected tropical diseases prevalent in Burma including filariasis, anthrax, Japanese encephalitis, and the emergent epidemic of Avian Influenza. What made this effort unique, and perhaps uniquely challenging, is that Burma/Myanmar was at the time, and remains at this writing, a deeply divided country, where scientific and humanitarian efforts have all too often been forced to choose between work "inside" the country and so with the approval or engagement of the ruling military junta, or "outside" the control of the junta, in partnership with non-Burman ethnic minority and democratic forces. As a measure of how divided the country can be, those on differing ends of the political spectrum do not agree on the name for country or her major cities and states. Those presenting data on Myanmar often have little accurate or current information on the border regions and may face government censorship over what data they do have – while groups working on the borders often know a great deal more about their areas of operation – but may be unwilling to openly divulge where and in what domains they are active for security reasons.

While all agree that Burma's peoples are in urgent need of health interventions and greatly expanded efforts to control and mitigate infectious diseases, the debate about how best to deliver those interventions has also been polarized, and there have been few, if any, opportunities for those engaged in the many and varied efforts underway to meet, share their efforts and undertakings, and discuss the potential for comprehensive responses. Given the politicization of humanitarian and health efforts in this troubled country, it seemed prudent to engage the many entities involved in a scientific meeting, where the diseases of importance could be addressed by the best available science and public health program approaches, and where health care providers working in challenging political environments might meet in a shared spirit of professionalism, mutual respect, and tolerance.

The conference was "off the record" to maximize the security of those most vulnerable, such as representatives of ethnic nationality health organizations whose political leaders have not signed cease-fire agreements with the ruling junta, and representatives from groups working under junta auspices in Burma proper, and so subject to surveillance, as is generally the case for Burmese professionals when they attend international meetings. Two exceptions were made to this rule: we agreed to a post-conference session with the press to share de-attributed outcomes with the lay media, and we offered to the speakers and participants that we would assist those interested in turning their talks into manuscripts for this special series in *Conflict and Health*. The papers presented here are among the core outcomes of the conference, and we are delighted to be able to present them to a wider audience.

## Infectious diseases in Burma and on her borders

What have we learned from bringing together the many players involved in Burma's health crisis? First, there is no debate that Burma's health care system is facing enormous difficulties and is currently unable to effectively respond to her health and humanitarian crisis. Malnutrition is widespread, and UNICEF estimates are that chronic malnutrition may affect up to a third of Burma's children, markedly increasing their susceptibility to infectious diseases. In 2000, Burma's health care system was ranked 190^th ^out of 191 nations by WHO[1]. Malaria is a major killer among infectious diseases, and Burma accounted for nearly half of all malaria deaths in the SEARO region (which includes India) despite having only a fraction of the regional population. Under-five childhood mortality was reported to be 106 per 1000 live births in 2006, compared to 21 per 1000 live births in Thailand and is known to be substantially higher in Eastern Burma's conflict areas[2]. These indicators are outcomes of the exceptionally low levels of health expenditure by the ruling State Peace and Development Council, or SPDC. UNICEF reported that SPDC spending on health care in Burma amounted to U.S. $0.40 cents per person per year in 2005, compared to U.S. $61 in neighboring Thailand[2]. There is a broad consensus on need within the country, and general recognition that the health crises of Burma have implications for her neighbors.

The regional impact of Burma's health crisis was addressed by speakers from Thailand, China, India, and Bangladesh. Examples of these challenges include the rising regional rates of MDR-TB and MDR-malaria. For both India and Thailand, the provinces with the highest rates of MDR-TB in their national programs were Burma border states. As Richards et al, point out in their malaria piece, the high prevalence of *p. falciparum *malaria in eastern Burma continues to serve as a large reservoir that likely constitutes a source of infection for neighboring countries. In addition, fake artesunates circulating in upper Burma's malaria zones have the potential to undermine the viability of this critical new class of agents[3]. In the context of HIV/AIDS, the Burma border zones of Yunnan in China, and Manipur and Nagaland in the Indian Northeast were all reported to be those countries most HIV – affected states and provinces. And in a strikingly similar and likely highly correlated interaction, Yunnan, the Indian Northeast, and Northern Thailand, all Burma border regions, were also the three nations most affected areas by another Burmese export – methamphetamines[4]. Dave Mathieson of Human Rights Watch reported at the conference that Burma accounts for roughly 25% of the amphetamines produced in Asia and that seizures in her neighbors had increased in 2006[3].

## The future

Taken together, these infectious disease realities underscored an obvious but critical message of the conference: infectious diseases do not respect man-made borders and political divisions – and single country approaches are unlikely to succeed in regional outbreaks. The case was made that this is particularly true for the unfortunate people of Burma, more than 1.2 million of whom have fled their homeland in recent years to seek work, food, security, and to escape conflict. With population flows of this magnitude, the unresolved health threats of Burma quickly become access to care issues for Burmese migrants and refugees in neighbor states, a reality highlighted by several speakers who provide health care services for these populations.

Despite these many challenges, a number of groups presented impressive program successes in difficult environments. Groups working inside Burma from cross-border approaches launched from Thailand into Eastern Burma, from Yunnan into the northern Burmese Kachin and Shan States, and those working in western Burma from the Indian Northeast reported on primary health care, reproductive health care, integrated malaria control, and HIV/AIDS efforts using cross-border approaches. Such efforts made it abundantly clear the "inside" vs "outside" distinction makes little sense when discussing these programs. They deliver services inside Burma to populations including internally displaced populations (IDPs) and families in cease-fire zones that are very much "inside" the country. The major distinction with these groups is that most do not operate under SPDC control or sanction – and so can reach populations not served by SPDC or its affiliates. A further distinction was found in data reported from the Mae Tao Clinic, which while on the Thai side of the Thai-Burma border serves an ever increasing proportion of Burmese from inside Burma proper who are neither migrants nor refugees – but health care seekers who come to Thailand for care unavailable or unaffordable at home. Patients from Burma accounted for some 47% of all Mae Tao Clinic attendees in 2005, including 72% of *p. falciparum *malaria cases, 75% of all patients requiring blood transfusions and 51% of all the clinic's HIV positive clients[5]. Burmese people are "voting with their feet" and making the long, arduous, and often dangerous journey to Thailand to seek health care.

Since the January conference Burma/Myanmar has seen the largest protests against military rule since the 1988 uprising: The Saffron Revolution of September 2007. Sparked initially by sharp rises in energy costs, which further impoverished an already threatened population, the non-violent uprising took on national scale when it was led by Burma's revered Buddhist monks[6]. The brutal crackdown which followed the uprising further isolated the military government, and brought heightened attention to the courage and the suffering of Burma's people. It also brought markedly increased calls for humanitarian assistance for the people of Burma, and numerous donors have responded with promised aid in humanitarian assistance and in health. While doubtless these efforts will save lives, it remains the case that Burma's humanitarian crisis is a man-made one: it is the direct outcome of military misrule, not simple poverty alone, and of the massive divestment in health and education, and in public sector spending more broadly, that has characterized the current regime of General Than Shwe and the SPDC. In addition to limiting spending on health care, the junta has also imposed tight restrictions on humanitarian assistance, and there is no evidence to date that these restrictions have eased in wake of Saffron Revolution. Tragically, the opposite seems to be the case: at this writing even more restrictive policy documents are circulating among NGOs in Rangoon, and the junta may make humanitarian assistance even more difficult to deliver through traditional channels[7]. Beyond these restrictions, ongoing forced displacement, forced labor, and other human rights violations continue to take their toll especially on the health status of ethnic minority border populations[8]. Cross-border approaches remain viable alternatives to access these most vulnerable border populations and those most likely to impact neighboring countries, but donor reluctance to support such efforts may hamper the ability of many groups to provide this assistance. In the short term, these realities suggest Burma will remain vulnerable to new and existing infectious disease threats – and her neighbors will continue to be challenged by the ongoing suffering of the Burmese people.

## Competing interests

The author(s) declare that they have no competing interests.
